# Phenotypic stability in scalar calcium of freshwater fish across a wide range of aqueous calcium availability in nature

**DOI:** 10.1002/ece3.7386

**Published:** 2021-05-02

**Authors:** Sarah Sanderson, Alison M. Derry, Andrew P. Hendry

**Affiliations:** ^1^ Redpath Museum and Department of Biology McGill University Montréal QC Canada; ^2^ Département des Sciences Biologiques Université du Québec à Montréal Montréal QC Canada

**Keywords:** convergence, countergradient variation, ecophysiology, environmental gradients, phenotypic buffering, water chemistry

## Abstract

Spatial environmental gradients can promote adaptive differences among conspecific populations as a result of local adaptation or phenotypic plasticity. Such divergence can be opposed by various constraints, including gene flow, limited genetic variation, temporal fluctuations, or developmental constraints. We focus on the constraint that can be imposed when some populations are found in locations characterized by low levels of an essential nutrient. We use scales of wild fish to investigate phenotypic effects of spatial variation in a potentially limiting nutrient—calcium. If scale calcium (we use “scalar” calcium for consistency with the physiology literature) simply reflects environmental calcium availability, we expect higher levels of scalar calcium in fish from calcium‐rich water, compared to fish from calcium‐poor water. To consider this “passive response” scenario, we analyzed scalar calcium concentrations from three native fish species (*Lepomis gibbosus*, *Percina caprodes*, and *Perca flavescens*) collected at multiple sites across a dissolved calcium gradient in the Upper St. Lawrence River. Contradicting the “passive response" scenario, we did not detect strong or consistent relationships between scalar calcium and water calcium. Instead, for a given proportional increase in water calcium across the wide environmental gradient, the corresponding proportional change in scalar calcium was much smaller. We thus favor the alternative “active homeostasis” scenario, wherein fish from calcium‐poor water are better able to uptake, mobilize, and deposit calcium than are fish from calcium‐rich water. We further highlight the importance of studying functional traits, such as scales, in their natural setting as opposed to only laboratory studies.

## Graphical abstract

We focus on the constraint that can be imposed when populations are found in locations characterized by low levels of an essential nutrient. We quantified scalar calcium concentrations from three native fish species collected at multiple sites across a dissolved calcium gradient in the Upper St. Lawrence River. We found that for a given proportional increase in water calcium across the wide environmental gradient, the corresponding proportional change in scalar calcium was much smaller.

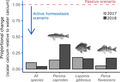

## 1 INTRODUCTION

Environmental gradients are expected to generate spatial variation in natural selection favoring different traits in populations living in different locations along the gradient (Endler, 1986; MacColl, 2011; Siepielski et al., 2017; Wade & Kalisz, 1990). One solution to this challenge is through genetically based local adaptation along the gradient as has been observed in many organisms (Hereford, 2009; Kawecki & Ebert, 2004). However, this solution can be difficult to achieve owing to constraints imposed by high gene flow, limited genetic variation, temporal fluctuations, or various trade‐offs (Brady, Bolnick, Angert, et al., 2019; Brady, Bolnick, Barrett, et al., 2019; DeFaveri & Merilä, 2014; Garant et al., 2007). An alternative solution under such constraints can be phenotypic plasticity, wherein individual organisms behaviorally, physiologically, or developmentally adjust their phenotypes to suit local conditions (Lande, 2009; Molina‐Montenegro et al., 2012; Sassenhagen et al., 2015; Schoeppner & Relyea, 2009). Yet this solution too can be difficult to achieve owing to developmental limits or constraints, time lags, imperfect cues, and various other factors (Auld et al., 2010; DeWitt et al., 1998; Hendry, 2016; Murren et al., 2015).

We expect that either solution (local adaptation or phenotypic plasticity) to an environmental gradient might be especially difficult when the environmental gradient is for a key limiting nutrient. In such cases, organisms at the lower end of the gradient might be unable, through either solution, to maintain optimal internal levels of those nutrients, resulting in observable deficits in those nutrients, which might then decrease health, vigor, performance, and fitness—perhaps to the point of extirpation. Obvious examples of nutrients that might cause such unsolvable limits would include the elements C, P, and N (Wetzel, 2001). Indeed, numerous examples have been reported where low levels of one or more of these elements have a dramatic effect structuring traits, populations, and communities (Cloern, 2001; Hessen, 1992; Smith, 2003). Additionally, inland herbivore populations have been reported to be limited by sodium (Na) (Kaspari et al., 2010; Welti et al., 2019). In aquatic systems, several additional nutrients can generate similar problems. For example, silicate is sometimes a key limiting nutrient determining the growth rate and biomass of diatoms and other phytoplankton (Kennington et al., 1999; Wassmann et al., 1999; Wu & Chou, 2003). Another example is that low concentrations of dissolved calcium ions can limit the growth rate—and hence population size and distribution—of freshwater organisms, such as crustaceans and fish (Baldwin et al., 2012; Cairns & Yan, 2009). Our study will focus on wild fish populations distributed across a gradient in this key element.

Interpreting organismal responses to environmental gradients benefits from considering multiple instances of organisms distributed across a given type of gradient (Langerhans & DeWitt, 2004). Similar phenotypic responses to the same type of gradient, commonly referred to as “parallelism,” are important observations bolstering the inference of cause‐and‐effect relationships. A common approach to studying such parallel evolution is to examine one species occupying the same environmental contrast in multiple locations (Oke et al., 2016). A limitation in this design is that variation among the replicates might simply reflect nonparallelism in the environments, and therefore in selection (Stuart et al., 2017). A less common approach—and the one we will here adopt—is to examine multiple species across the same environmental gradient in the same locations (Härer et al., 2018; Rosenblum et al., 2017). That is, we will consider the response of three fish species across the same gradient of dissolved calcium ions.

### 1.1 Calcium ions and scale physiology

Organisms selectively absorb elements, such as calcium, from their surrounding environment to incorporate them into biological structures and functions (Loewen et al., 2016). Calcium is an essential element involved in many physiological functions, including muscle fiber contraction, intracellular messaging, and reproduction (Crichton, 2008; Loewen et al., 2016). Calcium also serves as the foundation for biomineralization (Crichton, 2008). Although fish can uptake calcium from their diet, this source of calcium is minimal and they mostly acquire calcium directly from the surrounding water—mainly through their gills (Simkiss & Wilbur, 1989). Calcium uptake is achieved through branchial uptake via calcium channels in the apical membrane of chloride cells (Vanoevelen et al., 2011). Calcium is then deposited in the bones, scales, and skin (Lall & Lewis‐McCrea, 2007), with these components making up approximately 99% of the whole‐body fraction of calcium (Flik et al., 1986). In teleost fish, scales compose a particularly important part of body calcium in the form of calcium carbonate (CaCO_3_) and hydroxyapatite (Ca_5_(PO_4_)_3_(OH)) (Loewen et al., 2016; de Vrieze et al., 2012). For example, the amount of calcium in scales varies around 19%–24% dry weight in *Tilapia esculenta* (Garrod & Newell, 1958). In addition to their structural role, fish use their scales and bone as an internal calcium reservoir where they can store calcium for times of need, when it is mobilized and used for other cellular processes (Dacke, 1979).

We will focus on calcium in fish scales. Generally, fish scales are an important adaptive trait that can vary both intra‐ and interspecifically in response to a diversity of environmental gradients. Despite knowing that scales serve multiple purposes in the survival of fish, their diverse functional roles remain underexplored (Arendt et al., 2001). Examples include predator protection by acting as a protective layer (Bereiter‐Hahn & Zylberberg, 1993; Reimchen, 1988), swimming performance by reducing drag by breaking the boundary layer (Aleyev, 1977), and a calcium and phosphorus storage pool (Bereiter‐Hahn & Zylberberg, 1993). However, the adaptive relevance of trait variation in fish scales is relatively understudied in natural settings (Arendt et al., 2001; Flik & Verbost, 1993; Metz et al., 2014), and yet, scales provide a useful method of studying responses to environmental gradients through a parallel evolution approach because they are shared by all bony fishes (Helfman et al., 2009).

### 1.2 Objectives

Our objective was to compare how much (i.e., effect size) scale calcium, subsequently referred to as “scalar” calcium for consistency with the fish physiology literature, changed in relation to water calcium for three species of freshwater fish collected across an environmental gradient in aqueous calcium availability. We specifically investigated (a) whether low environmental levels of the essential nutrient calcium (Ca) was associated with lower levels of that element in essential anatomical features (scales), and (b) if the three fish species collected from shared locations had parallel responses (if any) of scalar calcium to water calcium. We considered two alternative scenarios in our predictions. The “passive response” scenario predicts higher calcium concentrations in scales at locations with higher calcium concentrations in the water. By contrast, the “active homeostasis” scenario predicts relatively constant scalar calcium across the water calcium gradient. Support for the latter scenario would suggest that selection favors—and fish are able to maintain—a narrow (perhaps “optimum”) range of calcium concentration in their scales despite dramatic spatial gradient in calcium availability in the water. No other studies that we are aware of have compared inter‐ and intraspecific variation in the scalar calcium of fishes across environmental gradients in natural settings, despite the importance of scales for survival and performance.

## 2 MATERIALS AND METHODS

### 2.1 Study system

**FIGURE 1:**
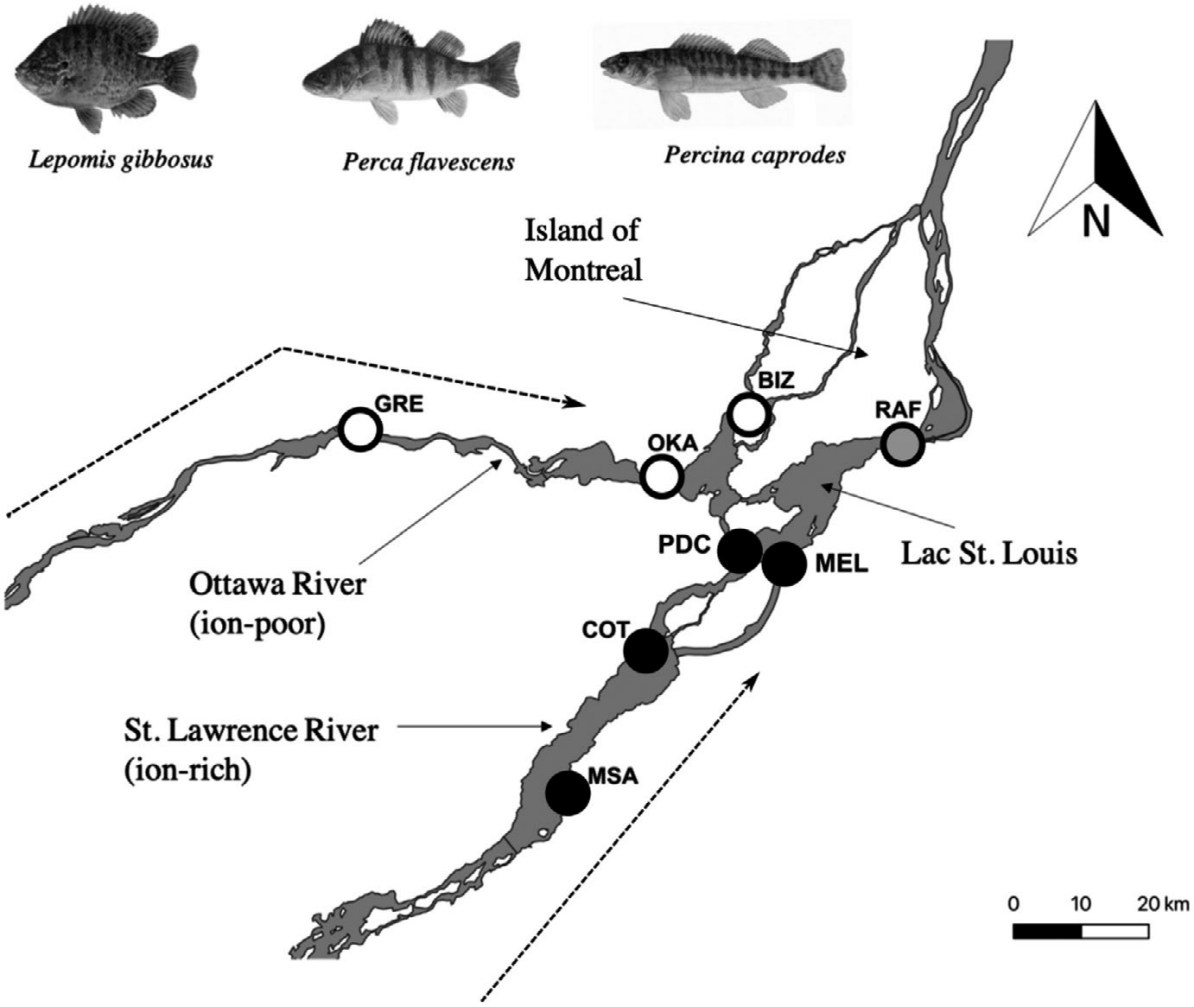
Map of study system, including Lac St. Louis, the Ottawa River and the St. Lawrence River. Ion‐rich water from the Great Lakes flows (flow direction is indicated by dashed arrows) from the southwest and ion‐poor water from the Ottawa River flows from the west, with the two water types meeting at the island of Montreal. Sites of fish and water sample collection are indicated by circles, where ion‐rich sites are indicated in black (MSA, COT, MEL, and PDC), ion‐poor sites in white (GRE, OKA, and BIZ), and the mixing zone site in gray (RAF)

Lac St. Louis (QC, Canada) (45° 22′N, 73° 81′W) is a large, spatially continuous freshwater ecosystem at the confluence of the Upper St. Lawrence River and the Ottawa River, and it is characterized by relatively strong environmental gradients between the north and south shores (Figure 1). These environmental gradients are created by “ion‐poor” water that enters Lac St. Louis from the Ottawa River (conductivity 70.5 μS/cm; dissolved Ca 9.8 mg/L) along its north shore and by “ion‐rich” water from the St. Lawrence River along its south shore (conductivity 363.0 μS/cm; dissolved Ca 46.1 mg/L). Conductivity was positively correlated (*R* ^2^ = 0.8) with dissolved calcium ion concentrations across this environmental gradient (Figure S1). Although there is some intra‐annual variation in water calcium with season and between years, the difference in water calcium between the water masses entering Lac St. Louis remain consistently greater than 19.2 mg/L (Astorg et al., 2021; Derry et al., 2013; Environnement et changements climatiques Canada, 2019, 2020; Kestrup & Ricciardi, 2010). The low concentrations of calcium in the Ottawa River relative to the St. Lawrence River compare with low calcium treatment levels used in many experimental settings of other studies (Baldwin et al., 2012; Iacarella & Ricciardi, 2015; Metz et al., 2014). In addition to conductivity and dissolved aqueous calcium, the two water masses in Lac St. Louis from the Ottawa River and the Upper St. Lawrence River, respectively, have other different environmental conditions, such as different concentrations of dissolved organic carbon (DOC; 3.64 mg/L on the south shore versus 19.70 mg/L on the north shore) and differences in food webs (Astorg et al., 2021). Research on acidification shows important effects on bone calcium concentrations, bone ossification, and eggshell thickness (Nybø et al., 1997; Ormerod et al., 1988, 1991). In our system, however, pH shows minimal variation and—in any case—pH levels are not in the range where large effects on calcium physiology in animals would be expected (pH = 8.6 in St. Lawrence water, pH = 8.0 in Ottawa River water; Table 2).

Calcium appears to be limiting to certain aquatic organisms in this ecosystem because, although native fish and macroinvertebrate species are distributed throughout the calcium gradient (Astorg et al., 2021), most Ponto‐Caspian invasive species (*Neogobius melanostomus, Echinogammarus ischnus*, and *Dreissena polymorpha*) are restricted to only the high‐calcium part of the environmental gradient (Astorg et al., 2021; Iacarella & Ricciardi, 2015; Jones & Ricciardi, 2005; Palmer & Ricciardi, 2005). Hence, the current distribution of these invasive species suggests that the Ottawa River serves as a natural uninvaded refuge for native species—likely due to dissolved ion concentrations. In short, the rich‐ and poor‐ion water masses of the Upper St. Lawrence River form an important continuous environmental gradient for several abiotic and biotic factors to which aquatic species might adapt.

### 2.2 Sample collection

**FIGURE 2:**
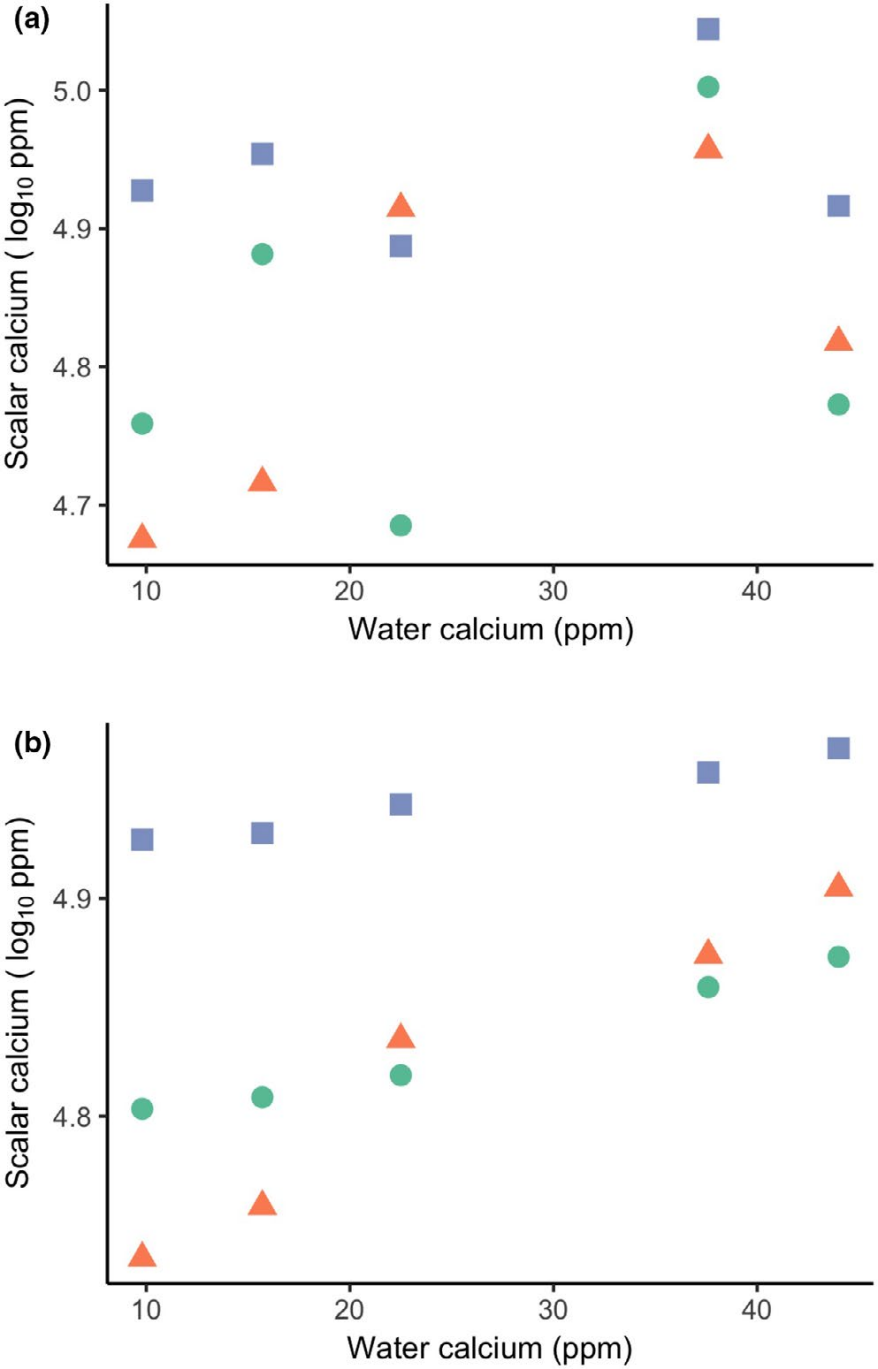
Mean scalar calcium (log_10_ transformed) of each species at every site along the water calcium gradient: (a) 2017 raw means and (b) 2017 fitted means obtained from the linear mixed effect model. Differences between the panels thus reflect the fact that the fitted means account for variation associated with other factors included in the statistical model. *L. gibbosus* fish are represented by triangles, *P. caprodes* by circles and *P. flavescens* by squares

**FIGURE 3:**
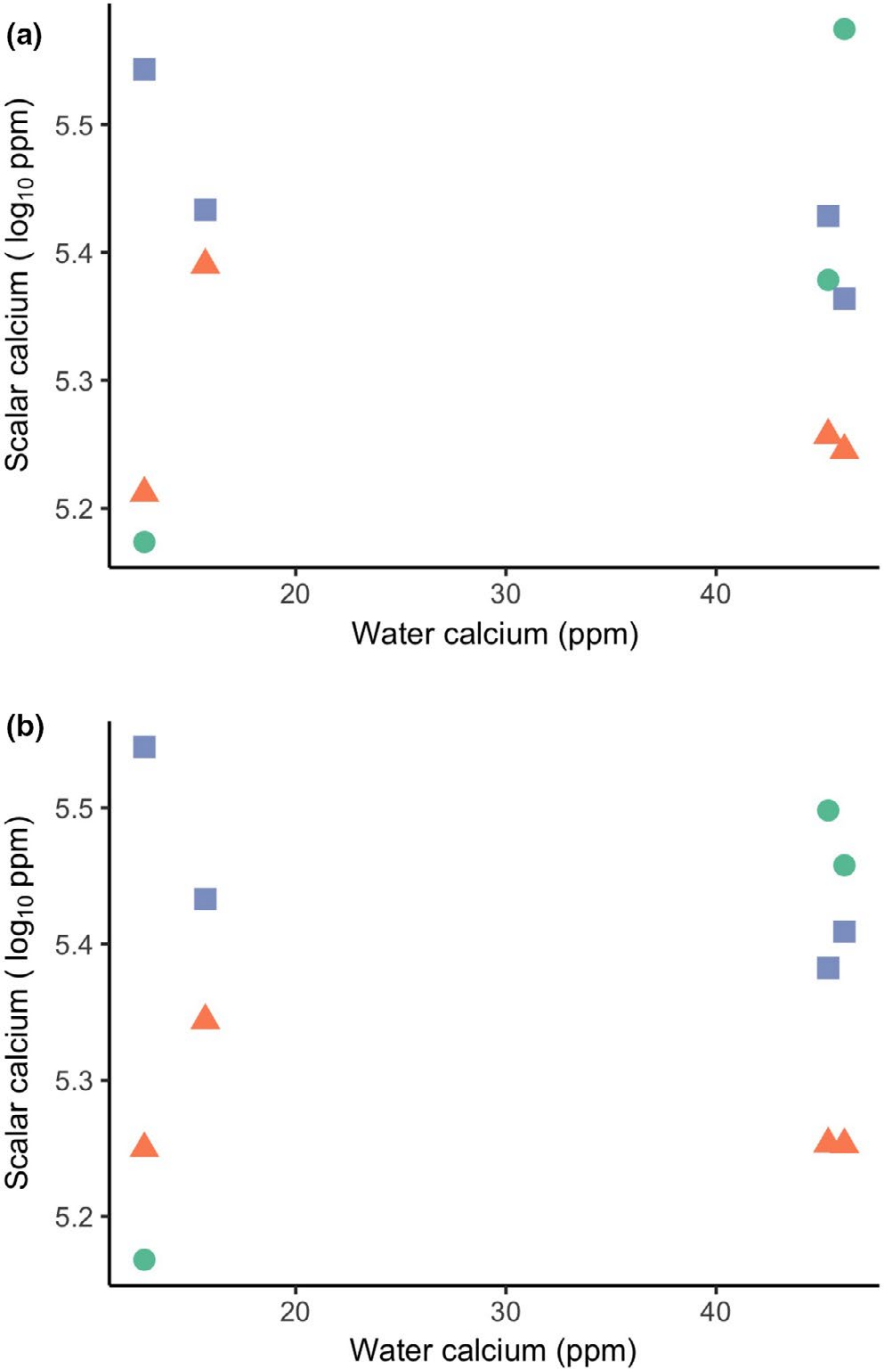
Mean scalar calcium (log_10_ transformed) of each species at every site along the water calcium gradient: (a) 2018 raw means and (b) 2018 fitted means obtained from the linear mixed effect model. Differences between the panels thus reflect the fact that the fitted means account for variation associated with other factors included in the statistical model. *L. gibbosus* fish are represented by triangles, *P. caprodes* by circles and *P. flavescens* by square

To answer our questions, we selected three different native fish species: Logperch (*Percina caprodes*), Pumpkinseed (*Lepomis gibbosus*), and Yellow Perch (*Perca flavescens*) (Bernatchez & Giroux, 2012). All three study species are *Perciformes: P. caprodes* and *P. flavescens* are part of the *Percidae* family whereas *L. gibbosus* is part of the *Centrarchidae* family. These specific species were selected because they are found throughout the environmental gradient, are fairly common throughout the area, and all occupy a mainly benthic habitat. In the summer of 2017, we collected fish across five different sites (PDC, MEL, RAF, BIZ, and GRE). When visually examining our results (Figures 2a,b, 3a,b), we noticed a possible relationship between water calcium and scalar calcium. Thus, in the summer of 2018, we expanded our sampling further up the environmental gradient to sample two sites in the ion‐poor water (GRE and OKA) and two sites in the ion‐rich water (COT and MSA). GRE was sampled both in 2017 and 2018 for a total of nine collections from eight sites. In total, 286 fish were collected from eight sites across the three species. All specimens were collected using a seine net deployed from shore (about 1 m water depth). The dimensions of the seine net were 114 cm by 407 cm with 5 mm mesh. The three target species were euthanized using tricaine methanesulfonate (MS222) at a concentration of 250–500 mg/L. Nontarget species were released immediately upon identification. Immediately after euthanasia, each fish was hung by its tail on a hook and put into a cooler for transportation to the laboratory, where they were transferred to a −20°C freezer. After 24 hr, the fish were taken off the hooks and transferred into plastic bags for storage. Fish collection and handling followed a McGill University animal care protocol #2016‐7831.

Physico‐chemical water measurements (temperature, °C; conductivity, μS/cm; dissolved oxygen, %; pH) were taken at each sampling site (Table 1) with a YSI series‐pro multi‐parameter sonde (model 10,102,030; Yellow Springs Inc., Yellow Springs, Ohio, USA). Dissolved calcium was measured using a Perkin Elmer Analyst 100 atomic absorption spectrophotometer at the McGill University Geotop laboratory, Montreal, QC, Canada. Water samples were collected in acid washed bottles and stored at 4°C until analyses for water chemistry at the GRIL (Groupe de Recherche Interuniversitaire en Limnologie) laboratory at Université du Québec à Montréal, Montréal, QC, Canada. Dissolved organic carbon (DOC) concentrations were measured in water samples filtered through of 0.45 μm filters (surfactant‐free membrane filters) after acidification (phosphoric acid 5%) followed by sodium persulphate oxidation using a 1,010 TOC analyser (O.I. Analytical, College Station, TX, USA). Total phosphorus (TP) was measured spectrophotometrically on the same instrument by the molybdenum blue method after persulphate digestion (Griesbach & Peters, 1991). Total nitrogen (TN; µg/L) was analyzed with a continuous flow analyzer (ALPKEM Flow Solution IV) using an alkaline persulfate digestion method, coupled with a cadmium reactor, following a standard protocol (Patton & Kryskalla, 2003).

**TABLE 1:** Physico‐chemical properties of the study sites recorded during summer 2017–2018

Site	Water type	Conductivity (μS/cm)	DOC (mg/L)	Ca^2+^ (mg/L)	pH	DO (%)	Temp (°C)	TP (μg/L)	TN (mg/L)
GRE	Ion‐poor	70.5	19.70	15.7	7.51	98.6	21.3	64.32	0.59
OKA	Ion‐poor	96.8	13.52	12.8	8.83	71.7	26.0	76.67	0.77
BIZ	Ion‐poor	80.7	6.79	9.8	7.59	89.3	20.0	42.94	0.50
RAF	Mixed	199.4	7.37	22.5	8.08	88.2	19.9	35.28	0.46
PDC	Ion‐rich	300.3	3.64	44.0	8.40	98	18.9	18.60	0.41
MEL	Ion‐rich	307.3	19.62	37.6	8.50	108.5	20.8	66.41	0.44
COT	Ion‐rich	263.8	4.01	45.3	8.80	119.7	26.0	11.34	0.33
MSA	Ion‐rich	363.0	6.04	46.1	8.87	61.9	26.5	15.40	0.40

Abbreviations: Ca^2+^, dissolved calcium ions; DO, dissolved oxygen, Temp, temperature; DOC, dissolved organic carbon; TP, total phosphorus and TN, total nitrogen.

### 2.3 Mineral analyses

We removed 30 scales from the left flank at the mid‐lateral region of every fish and dried them overnight at 80°C (Jeziorski & Yan, 2006; Metz et al., 2014) for mineral analysis. The dried scales were dissolved in 60–100 μl of 65%–69% nitric acid for 24 hr. The 2017 and 2018 samples were then analyzed using different methods. The reason was that promising results in the 2017 data encouraged us to increase precision for the 2018 samples and to also estimate scalar phosphorus concentrations. Owing to the different analytical methods, we do not make any direct interannual comparisons of scalar calcium levels. Rather, we treat the two years of data as replicate tests of the same hypothesis. The reason for phosphorus analyses is that calcium is stored in scales in the form of calcium carbonate and hydroxyapatite which includes phosphorus. The 2017 samples were diluted in 4–8 ml of nanopure water and analyzed by Atomic Absorption at a wavelength of 422.7 nm using a Perkin Elmer Analyst 100 atomic absorption spectrophotometer at McGill University Geotop (Research Centre on the Dynamics of the Earth System). Scales from 2018 samples were dried overnight and dissolved in 500 μl of nitric acid and diluted in 10 ml of nanopure water. 2018 samples were analyzed with ICP‐OES (Inductively Coupled Plasma Optimal Emission Spectrometry) using a Thermo Fisher Scientific iCAP 6,500 Duo avec Autosampler CETAC ASX‐520 to get both scalar calcium and phosphorus concentrations. Calcium was analyzed at wavelengths of 422.7 nm and 431.865 nm, whereas phosphorus concentrations were analyzed at wavelengths of 185.891 nm and 214.914 nm.

### 2.4 Data analyses

Our main goal was to investigate the relationship between scalar calcium and environmental ion concentrations in nature, thus informing whether fish adjusted calcium deposition in their scales in response to the environmental ion gradient. Data from each summer (2017 and 2018) were analyzed separately because they represented two independent tests of the same question and because they involved different spatial scales and scalar calcium methodology (Figure 2a,b; Table 2). For all analyses, scalar calcium and body size (total length) data were log‐transformed to better meet the assumptions of a Gaussian distribution (Bland & Altman, 1996; LaBarbera, 1989).

**TABLE 2:** Linear mixed effect models predicting scalar calcium concentrations of *P. caprodes* (PC), *L. gibbosus* (LG) and *P. flavescens* (PF) along the ionic gradient. Scalar calcium concentrations and body size were log_10_ transformed before model fitting. Significance of effects was estimated using a log‐likelihood ratio test.

Effect	Estimate (±SE)	*df*	LLR	*p*‐value
2017 (marginal *R* ^2^ = 0.09, conditional *R* ^2^ = 0.20)^a^				
Intercept	5.15 (0.35)	173		
SpeciesLG	−0.07 (0.09)	173	15.24	**.004**
SpeciesPF	0.15 (0.08)	173		
WaterCa	0.002 (0.003)	3	2.22	.528
Body size	−0.19 (0.18)	173	1.61	.281
SpeciesLG * waterCa	0.003 (0.003)	173	1.29	.522
SpeciesPF * waterCa	−0.0008 (0.003)	173		
Random (repeatability)^b^	0.12			
Site	0.073		7.008	**.008**
Residual	0.195			
2018 (marginal *R* ^2^ = 0.22, conditional *R* ^2^ = 0.22)^a^				
Intercept	5.94 (0.38)	94		
SpeciesLG	0.19 (0.16)	94	22.81	**.0004**
SpeciesPF	0.53 (0.16)	94		
WaterCa	0.01 (0.003)	2	11.77	**.008**
Body size	−0.54 (0.21)	94	6.71	**.01**
SpeciesLG * waterCa	−0.008 (0.004)	94	10.22	**.006**
SpeciesPF * waterCa	−0.013 (0.004)	94		
Random (repeatability)^b^	1.06e^−9^			
Site	6.98e^−6^		2.41e^−8^	.10
Residual	0.22			

Bold values represent significant *p*‐values.

^a^ Marginal *R* ^2^ describes the proportion of total variance explained by the fixed effects in the model. Conditional *R* ^2^ described the proportion of total variance explained by the fixed effects and the random effect together.

^b^ Repeatability was estimated as the proportion of the remaining variance (not explained by the fixed effects).

We examined the extent to which scalar calcium varied along the ionic gradient by analyzing our data in linear mixed effect models where scalar calcium was the response variable. Fixed effects included water calcium, species (*P. caprodes*, PC; *L. gibbosus*, LG; and *P. flavescens*, PF), and body size (L_s_). The random effect was sampling site in a given year. The significance of each fixed effect was tested by comparing the log‐likelihood ratios of the full model described above to a model lacking each fixed effect (Zuur et al., 2009). Post hoc analyses were performed to determine which species were different from each other.

Given that the species‐by‐water calcium interaction was sometimes significant (Table 2) in the full models described above, we also constructed species‐specific models. Such species‐specific models (Table S1) allowed us to determine the relationship between scalar calcium and water calcium for each species, and if it showed a parallel response.

To go beyond statistical significance (as above) to also estimate the *importance* of the water calcium gradient to scalar calcium, we used an effect size measure akin to elasticity or “proportional sensitivity” (Link & Doherty, 2002) that compares the ratio of proportional change between two variables across the same sites:

$$\text{Ratio of proportional changes}=\frac{\left|\ln\left(y_{2}\right)‐\ln\left(y_{1}\right)\right|}{\left|\ln\left(x_{2}\right)‐\ln\left(x_{1}\right)\right|}$$

where *y*_1_ and *y*_2_ are the predicted estimates of scalar calcium concentrations obtained from fixed values of water calcium *x*_1_ and *x*_2_ in the linear mixed effect models described above. (Note: This is equivalent to the slope of a log–log relationship between scalar calcium and water calcium.) If fish are passively up‐taking calcium ions from the water and depositing them in their scales (“passive response”), then a proportional equivalence should be observed between increasing water calcium and increasing scalar calcium. That is, for 10% increase in water calcium along the environmental gradient, we should observe a 10% increase in scalar calcium, which would correspond to a ratio of proportional change of 1. Alternatively, if fish are regulating their calcium uptake within a specific range despite highly variable calcium availability in the environment (“active homeostasis”), we should observe that a given proportional increase in water calcium should lead to a correspondingly smaller proportional increase in scalar calcium: that is, a ratio of proportional change of <1.

All statistical analyses were performed in R version 3.6.1 (R Core Team, 2019). All linear models were performed with lme using REML estimation in the *nlme* package (Pinheiro et al., 2019). We assumed a Gaussian error distribution which we confirmed by visual inspection of the residuals of the model and QQ plots (Zuur et al., 2009). We obtain R^2^ values using the *r.squaredGLMM* function in the *MuMIn* package (Bartoń, 2019). Post hoc comparisons were obtained using the *pairs* function, which compares the estimated marginal means, in the *emmeans* package (Lenth, 2019).

## 3 RESULTS

We did not observe a consistent relationship between scalar calcium and water calcium for all three freshwater fish species caught in the wild. Fitted values of the 2017 dataset suggested that fish in ion‐poor water have lower scalar calcium—but the trend is nonsignificant (Figure 2b; Table 2). Moreover, when we expanded sampling along the ionic gradient to include sites at the extremes of the gradient (2018 dataset), we no longer observed any indication of a general relationship between scalar calcium and water calcium (Figure 3b). In the 2017 dataset, more water calcium was not a significant predictor of more scalar calcium (*p* = .53), whereas it was a significant predictor in the 2018 dataset (*p* = .008). Even in this case of this statistically significant effect, however, the association was very weak—as described below.

**FIGURE 4:**
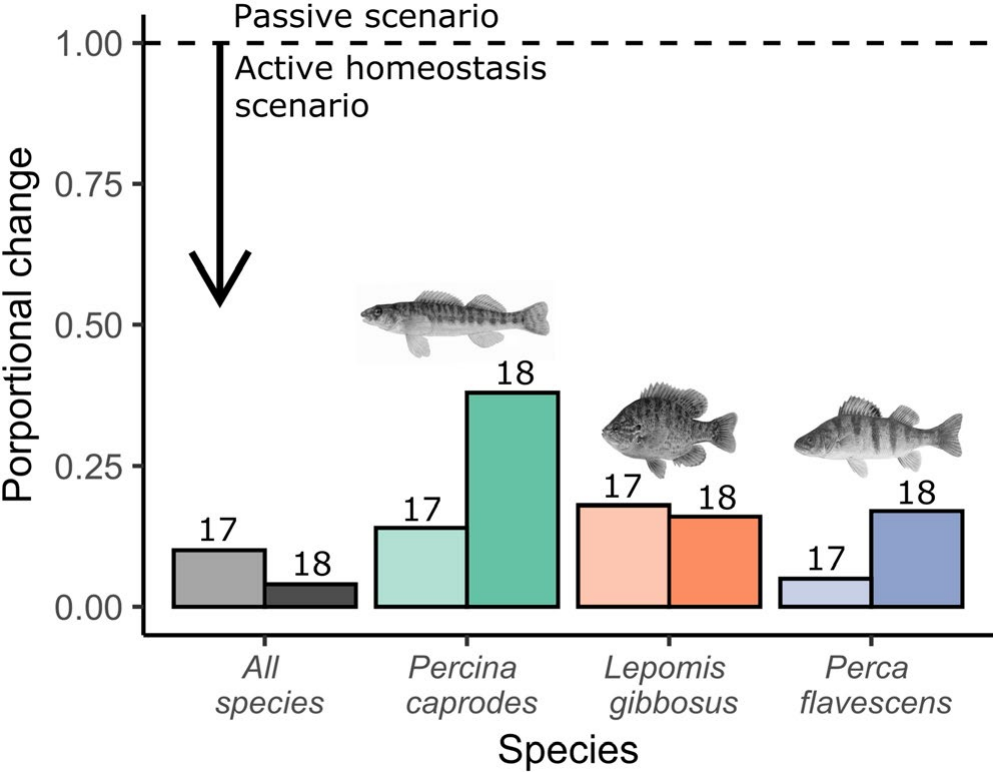
Proportional differences in scalar calcium concentration across the study sites in relation to differences in water calcium concentrations across those sites. Proportions were obtained using the modified equation for elasticity presented in the Methods. 2017 data is represented by 17. 2018 data is represented by 18

In comparing between‐species responses, post hoc analyses suggest that in both years *L. gibbosus* and *P. flavescens* differed from each other (2017, *p* = .002; 2018, *p* = .002), *P. caprodes* and *L. gibbosus* did not differ from each other (2017, *p* = .99; 2018, *p* = .65, and *P. caprodes* and *P. flavescens* differed in 2017, but not in 2018 (2017, *p* = .004; 2018, *p* = .14). Given that the species‐by‐water calcium interaction was sometimes significant (Table 2), we also constructed species‐specific models. In all but one instance, water calcium was not a significant predictor of scalar calcium (Table S1); the exception being 2018 *P. caprodes*. However, in that case, we had small sample sizes and were lacking any samples for one of the sites (GRE) (Table S2). For the proportional relationship (i.e., elasticity) between water calcium and scalar calcium (Figure 4), we obtained an average value of 15%. Thus, for a given proportional increase in water calcium across the environmental gradient, we recorded a much smaller proportional change in scalar calcium (see partial *R* ^2^ values obtained from the linear mixed models; Figure 4). These effect sizes confirm that fish regulate their scalar calcium within a much narrower range than the range of calcium availability in the water.

Total water phosphorus across the entire study area varied between 11.34 and 76.67 μg/L (Table 1), which is in the mesotrophic to eutrophic range of productivity (Wetzel, 2001). Water phosphorus availability thus should not be limiting for fish. Moreover, we found a linear relationship between scalar calcium and scalar phosphorus for each of the three species (Figure S2), suggesting no phenotypic variation particular to phosphorus.

## 4 DISCUSSION

Calcium limitation in aquatic systems has the potential to be an important selective factor shaping adaptive responses in fish scalar calcium, which we examined for three sympatric species across the same environmental gradient in water calcium. We found that all three freshwater species (*P. caprodes*, *L. gibbosus*, and *P. flavescens*) maintain a relatively narrow range of calcium in their scales despite the strong gradient in water calcium. Relationships between scalar calcium and water calcium were—even if statistically significant in a few instances—inconsistent and always very weak (Figures 2a,b, 3a,b; Table 2; Table S1). Thus, our results are consistent with the “active homeostasis” scenario rather than the “passive response” scenario outlined in the Introduction. This outcome shows that fish across the ion gradient possess physiological mechanisms that allow the uptake of sufficient calcium from their environment to meet their needs. This result suggests that some minimal level of scalar calcium is important for individuals of all three species that likely resulted from natural selection on heritable differences. At the other end of the spectrum, fish in calcium‐rich environments clearly do not continue to deposit more and more calcium in their scales, suggesting a possible constraint to having too much calcium in scales. The *why* (natural selection) and the *how* (evolution or plasticity) of these two outcomes will be unpacked in the following sections. As our study is the first to explore scalar calcium across environmental gradients in nature from both an intraspecific and interspecific perspective, we hope that these discussions help to motivate and guide additional work.

### 4.1 Why do fish in calcium‐poor environments maintain relatively high scalar calcium?

Our finding that all three fish species did not show appreciably lower scalar calcium in calcium‐poor water suggests that selection favors enhanced calcium uptake and deposition under calcium‐poor conditions. One selective force might be that fish seek to maintain relatively high levels of scalar calcium as a storage reservoir for future use (Arendt et al., 2001). Indeed, calcium can be resorbed from the scales and mobilized into its ionic form to be used in other functions such as metabolic demand and physiological activity (Metz et al., 2014). Another reason to maintain scalar calcium levels might be protection from predators. For instance, higher concentrations of scalar calcium result in thicker scales which should, in turn, increase protection from puncture (Arendt et al., 2001). Supporting this idea, predation has been suggested to cause the evolution of thicker plates and longer spines in threespine stickleback, even in calcium‐poor environments. Bell et al. (1993) found that, in calcium‐poor environments, stickleback populations did not have pelvic (bone) reduction when predation was high but did have pelvic reduction when predation was low. However, the fitness costs that underpin pelvic reduction in calcium‐poor water remain unclear (Rollins et al., 2014). In our study system, predation pressure is likely high in both water types given the nearly ubiquitous presence of native predators (Astorg et al., 2021). In particular, *P. caprodes*, *L. gibbosus*, and *P. flavescens* in both water types are known to be important prey for many predators, including *Micropterus dolomieu* (Smallmouth Bass), *Esox lucius* (Northern Pike), *Sander canadensis* (Sauger), and *Esox masquinongy* (Muskellunge) (Bernatchez & Giroux, 2012). Further study will be required to establish the particular mechanisms (e.g., predation or storage or both or something else) maintaining high scalar calcium even in calcium‐poor water in our study system (Table 3).

**TABLE 3:** Partial *R* ^2^ calculated by removing the effect of interest from the model

	2017		2018	
	*R* ^2^ marginal	*R* ^2^ conditional	*R* ^2^ marginal	*R* ^2^ conditional
Full model	0.09	0.20	0.22	0.22
Species	0.02	0.12	0.04	0.04
Water Calcium	0.07	0.17	0.14	0.15
Interaction (species*water calcium)	0.09	0.19	0.15	0.15

### 4.2 Why do fish in calcium‐rich environments not deposit even more calcium in their scales?

If fish in calcium‐poor water experience strong natural selection for improved calcium uptake and deposition, why then do fish in calcium‐rich water not simply deposit even more calcium in their scales? The fact that fish in these latter environments do not have higher scale calcium suggests that having too much calcium in scales has negative effects on the scales themselves, or on some other trait or suite of traits. Although the functional properties of scales are not fully understood, they are argued to be diverse and might therefore generate trade‐offs. For instance, thick and calcium‐rich scales should increase skin stiffness, which should decrease swimming efficiency in at least some contexts (Long et al., 1996; McHenry et al., 1995; Wainwright & Lauder, 2016). In particular, skin stiffness determines the translation efficiency of muscle contraction to forward movement (McHenry et al., 1995). Alternatively, maintaining high levels of scalar calcium might trade‐off with some important nonscale function, such as growth or reproduction. For instance, it has been shown in multiple species of fish, including *L. gibbosus*, that a trade‐off exists between scale strength, which is correlated with scalar calcium, and body growth (Arendt et al., 2001; Flik et al., 1986; Flik & Verbost, 1993). Moreover, Flik and Verbost (1993) found that adding growth hormones to water with low calcium concentrations caused fish to decrease the amount of calcium found in their scales. Such a scalar calcium versus growth trade‐off might imply that, past a certain level of scalar calcium concentrations, it would be more profitable to invest any additional calcium in increasing growth or some other biological function. For instance, scalar calcium concentrations could be trading‐off with metabolic demand. In fact, calcium can be re‐allocated to metabolic functions, such that fast‐growing individuals might require higher metabolic calcium levels simply to meet increased metabolic rates (McCarthy, 2000).

### 4.3 How do fish maintain the narrow range of scalar calcium?

We have just argued that fish—in our study system at least—are under selection to maintain a “baseline” level of calcium in their scales but, past this “baseline,” more calcium becomes costly or less important than investing extra calcium in other physiological processes. The resulting narrow range of scalar calcium despite the large water calcium gradient could reflect some combination of adaptive plasticity and genetic divergence (i.e., local adaptation). Although we cannot rule out a contribution of plasticity, it is noteworthy that the patterns we observed are not consistent with plastic responses seen in laboratory studies. For instance, fish held in varying concentrations of water calcium maintain their plasma Ca^2+^ levels, but do not maintain scalar calcium concentrations (Flik et al., 1986; Shephard, 1981). In fact, when exposed to low levels of water calcium in the laboratory, both tilapia and zebrafish decrease calcium concentrations in their scales (Flik et al., 1986; Metz et al., 2014). However, these previous studies were short term (e.g., days or weeks). Thus, to fully explore the potential role of plasticity, one would have to raise fish for longer periods of time in different levels of water calcium.

With respect to genetic divergence, we can certainly point to previous work on other systems that has shown a strong genetic basis for variation in calcium uptake, regulation, and deposition. Calcium ion transport across the branchial membrane is mediated through Ca^2+^ ATPase (Marshall et al., 1995; Perry & Flik, 1988; Verbost et al., 1994), and the activity level of this enzyme is correlated with the amount of plasma calcium levels (Shephard, 1981). Moreover, in teleosts, calcium is regulated through PTH hormones and related proteins (Rotllant et al., 2005). Teleosts have two forms of PTH hormones, two forms of PTHrP proteins, and one PTH intermediate protein which are all encoded by separate genes (Guerreiro et al., 2007). The key question is whether genetic divergence among environments contributes to differential expression of these genes. Although little work has been done in this area, studies of stickleback fishes have shown strong parallel divergence between populations in calcium‐related traits and genes (Gibbons et al., 2016; Spence et al., 2012). For instance, Gibbons et al. (2016) found that freshwater populations have a significantly higher gene expression of epithelial calcium channels (ECaC) at the gill—used to pump calcium from the environment into plasma—than do marine populations. However, we are not aware of studies that have directly tested for local adaptation in calcium transport, regulation, or deposition among populations of the species that we studied.

Given all of the above, we postulate that the plastic effect of low water calcium in reducing scalar calcium is counteracted through genetic compensation that increases calcium uptake, regulation, and deposition. The resulting stability of scalar calcium in wild fish across the gradient corresponds to a pattern termed “countergradient variation” (Conover et al., 2009; Conover & Schultz, 1995). We do not yet have direct confirmation of this scenario. However, genetic divergence among our study populations does seem possible given that our upstream‐most sites are very distant from calcium‐rich water, suggesting that gene flow constrains would be unlikely (Endler, 1986; Richardson et al., 2014; Urban et al., 2020)—at least at the extremes of the gradient. Moreover, research on other freshwater fishes has shown the prevalence of genetic divergence over small spatial scales, such as in stickleback (e.g., Baumgartner, 1986; Bell, 1982; Berner et al., 2008; McPhail, 1984). Indeed, the few studies conducted on fish across the same gradient in our study system show some divergence in a number of traits. For example, Lake Sturgeon (*Acipenser fulvescens*) from Lac des Deux Montagnes (Ottawa River) and Lac St. Louis (St. Lawrence) differ morphologically from each other (Guénette et al., 1992). Moreover, studies of other organisms across the same gradient have confirmed genetic divergence in some phenotypic traits and a countergradient plastic response in some life‐history traits. For instance, Derry et al. (2013) found strong plastic effects on postmoult calcification in amphipods (more rapid in ion‐rich water) and genetic differences in multiple life‐history traits in Lac St. Louis, the same study system addressed by this paper.

Further research will be necessary to confirm or refute the ideas outlined above. For example, plasticity and countergradient variation could be tested by reciprocal transplant experiments or common garden experiments. If fish maintain scalar calcium concentrations through a plastic response, we would expect all individuals (or populations) to maintain stable scalar calcium concentrations when exposed to calcium‐poor water. Alternatively, genetic compensation leading to countergradient variation would be inferred if fish from calcium‐poor water populations maintain higher scalar calcium than do fish from calcium‐rich water populations, when both are raised in common (especially poor) calcium water. Finally, gene expression studies would indicate which genes might be involved in differential responses to calcium, and genomic analyses would indicate if those genes had corresponding divergence in their sequences. Although some systems are “even more ion‐poor,” previous studies have used the terms “ion‐poor” and “ion‐rich” highlighting the important contrast in calcium availability between the two water bodies (Astorg et al., 2021; Derry et al., 2013). Future studies could focus on fish collected from ecosystems with more extreme calcium limitation (e.g., freshwater lochs on North Uist, Scotland (Giles, 1983)) and/or in ecosystems that have experienced recent declines in water calcium (e.g., associated with forest harvesting in the watershed; (Jeziorski et al., 2008)). Nonetheless, Lac St Louis presents excellent opportunities for testing evolutionary and ecological mechanisms in understanding fish scalar responses to calcium gradients because of the interplay of both strong selection and spatial connectivity in this river system.

### 4.4 Additional implications

All of the native species that we studied were capable of maintaining high scalar calcium even in calcium‐poor water, and all of them were abundant in that water (Figures 2a,b, 3a,b; Table S2). These patterns suggest that a particular scalar calcium level is important to fish—serving as a key functional trait—and that all of the studied native fishes could achieve this level, perhaps through adaptive plasticity (also called “phenotypic buffering” (Reusch, 2014)) or genetic compensation (leading to countergradient variation (Grether, 2005)). Yet not all fish in the same area can do this. Invasive Round Gobies (*Neogobius melanostomus*), for example, are not found in the Ottawa River, and are very rare in the plume of Ottawa River water after it joins the St. Lawrence River (Iacarella & Ricciardi, 2015). These gobies also show low foraging, growth, and survival rates when held in calcium‐poor water in the laboratory (Baldwin et al., 2012; Iacarella & Ricciardi, 2015). This putative adaptive failure could reflect high gene flow constraining local adaptation, low genetic variation in calcium‐relevant genes, insufficient time for adaptation, or some combination of these factors. We therefore suggest that studies of important physiological traits expressed in native fishes across environmental gradients that invasive fish cannot tolerate, can be a guide to identifying the specific factors, and perhaps genes, that mediate the spread of aquatic invasive species.

## CONFLICT OF INTEREST

The authors declare no conflict of interest.

## AUTHOR CONTRIBUTIONS

**Sarah Sanderson:** Conceptualization (lead); Data curation (lead); Formal analysis (lead); Investigation (lead); Methodology (lead); Validation (lead); Visualization (lead); Writing‐original draft (lead); Writing‐review & editing (equal). **Alison Derry:** Conceptualization (equal); Funding acquisition (equal); Resources (lead); Supervision (equal); Writing‐original draft (equal); Writing‐review & editing (lead). **Andrew Hendry:** Conceptualization (equal); Funding acquisition (equal); Methodology (equal); Project administration (lead); Resources (equal); Supervision (lead); Writing‐original draft (equal); Writing‐review & editing (lead).

## ETHICAL APPROVAL

Fish collection and handling followed a McGill University animal care protocol #2016‐7831.

## Supporting information

Supplementary Material

## Data Availability

Data are available from the Dryad Digital Repository: https://doi.org/10.5061/dryad.1ns1rn8t1
