# Supplementary material for: Phenotypic stability in scalar calcium of freshwater fish across a wide range of aqueous calcium availability in nature
Source: Ecol Evol. 2021 May 2;11(11):6053–65. doi: 10.1002/ece3.7386 (PMC8207426; doi:10.1002/ece3.7386)
Supplement: Supplementary file 1 — Supplementary Material [file ECE3-11-6053-s001.docx]

**APPENDIX**

**FIGURES**


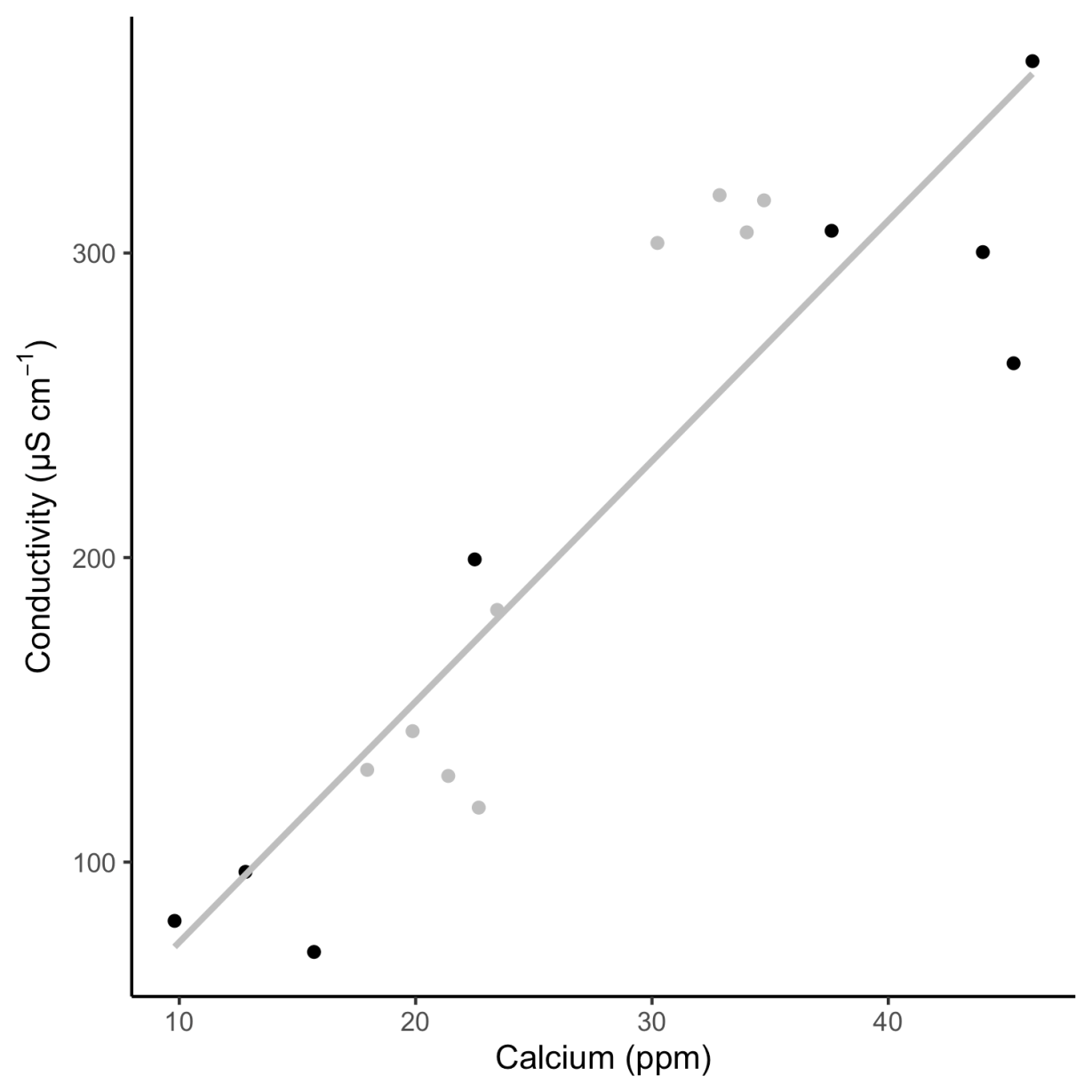


**FIGRUE A1** Linear relationship between conductivity (μS cm^-1^) and dissolved calcium ions (ppm) in the study system where the R^2^ = 0.81 and p-value = 9.069e-7. Black symbols indicate the eight study sites and gray symbols indicate additional sites in the study system from Astorg *et al.* (2020).

#### **
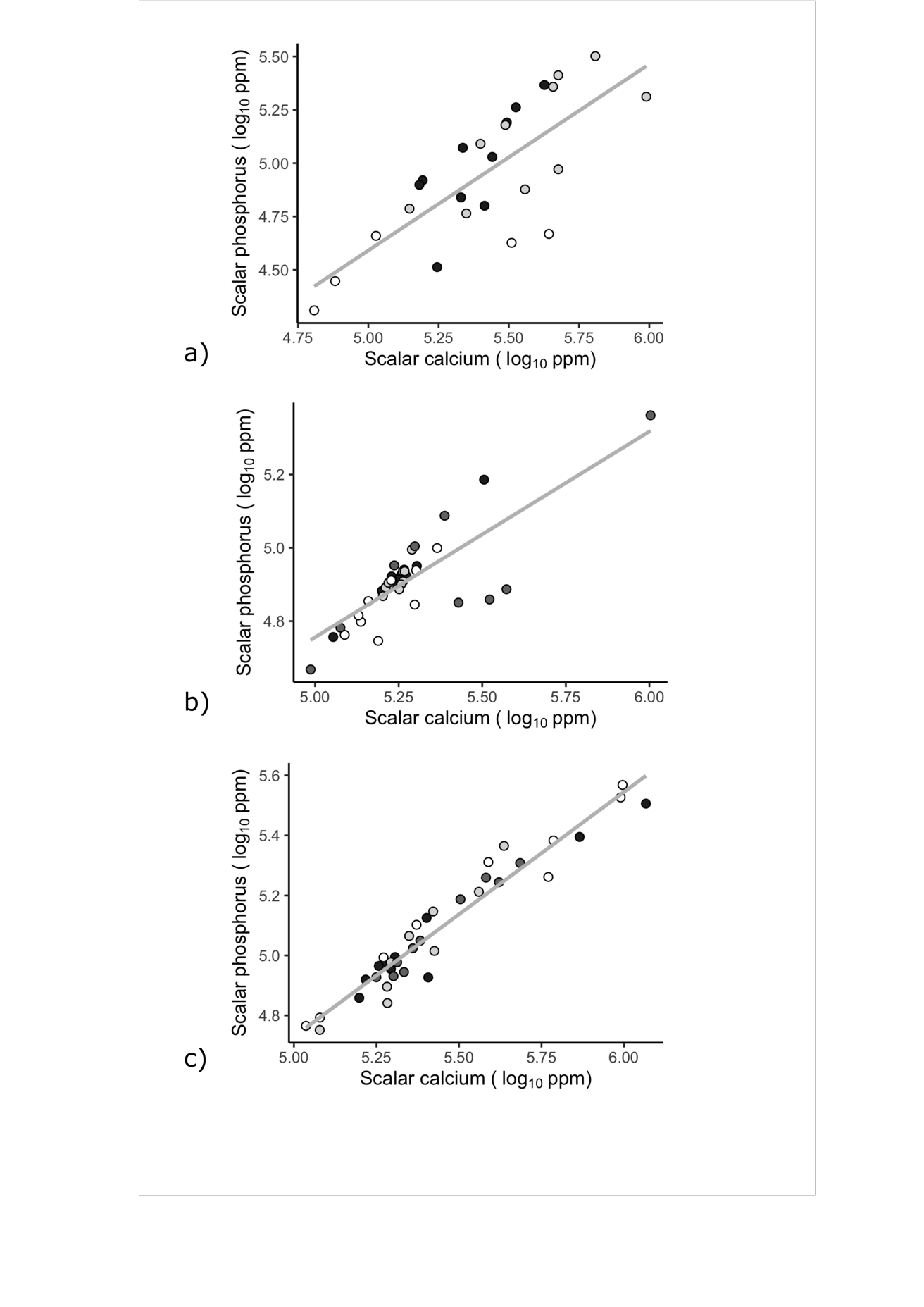
FIGURE A2** Linear relationship between scalar calcium concentrations and scalar phosphorus concentrations for 2018 samples where a) *P. caprodes* (R^2^ value = 0.58; p-value = 1.35e^-05^) b) *L. gibbosus* (R^2^ = 0.74; p = 2.43e^-12^) c) *P. flavescens* (R^2^ = 0.93; p <2.2e^-16^). Black symbols are fish from COT, dark gray symbols are fish from GRE, light gray symbols are fish from MSA, and white symbols are fish from OKA.

##### **TABLES**

##### **TABLE A1** Linear mixed effect model predicting scalar calcium concentrations of *P. caprodes*, *L. gibbosus,* and *P. flavescens* along an ionic gradient in the St. Lawrence system. Scalar calcium concentrations and body size were log_10_ transformed before model fitting. Significance of effects were estimated using a log-likelihood ratio test.

| **Effect** | **Estimate** | **d.f.** | **LLR** | **p-value** |
| --- | --- | --- | --- | --- |
| *2017 P. caprodes (marginal R^2^=0.19, conditional R^2^=0.44)^†^* | | | | |
| Intercept | 0.765 (0.981) | 53 |  |  |
| Water calcium | 0.006 (0.005) | 3 | 1.95 | 0.16 |
| Body size (L_s_) | 2.07 (0.05) | 53 | 14.19 | **0.0002** |
| **Random**  (repeatability)^‡^ | 0.31 |  |  |  |
| Site | 0.141 |  | 5.94 | **0.01** |
| Residual | 0.211 |  |  |  |
| *2017 P. flavescens (marginal R^2^=0.02, conditional R^2^=0.2)^†^* | | | | |
| Intercept | 5.46 (0.584) | 62 |  |  |
| Water Calcium | 0.0009 (0.002) | 3 | 0.25 | 0.62 |
| Body size (L_s_) | -0.27 (0.29) | 62 |  |  |
| **Random**  (repeatability)^‡^ | 0.18 |  | 0.72 | 0.40 |
| Site | 0.06 |  | 4.11 | **0.04** |
| Residual | 0.13 |  |  |  |
| *2017 L. gibbosus (marginal R^2^=0.14, conditional R^2^=0.23)^†^* | | | | |
| Intercept | 5.62 (0.44) | 50 |  |  |
| Water calcium | 0.003 (0.003) | 3 | 1.42 | 0.23 |
| Body size (L_s_) | -0.47 (0.22) | 50 | 5.23 | **0.02** |
| **Random**  (repeatability)^‡^ | 0.12 |  |  |  |
| Site | 0.07 |  | 1.31 | 0.25 |
| Residual | 0.19 |  |  |  |
| *2018 P. caprodes (marginal R^2^=0.16, conditional R^2^=0.49)^†^* | | | | |
| Intercept | 6.64 (1.95) | 21 |  |  |
| Water calcium | 0.01 (0.008) | 1 | 3.9 | **0.05** |
| Body size (L_s_) | -0.963 (1.070) | 21 | 0.55 | 0.70 |
| **Random**  (repeatability)^‡^ | 0.39 |  |  |  |
| Site | 0.19 |  | 1.5 | 0.22 |
| Residual | 0.24 |  |  |  |
| *2018 P. flavescens (marginal R^2^=0.19, conditional R^2^=0.19)^†^* | | | | |
| Intercept | 6.89 (0.52) | 34 |  |  |
| Water calcium | -0.002 (0.002) | 2 | 0.51 | 0.47 |
| Body size (L_s_) | -0.78 (0.30) | 34 | 6.87 | **0.01** |
| **Random**  (repeatability)^‡^ | 3.97e^-8^ |  |  |  |
| Site | 47.38e^-06^ |  | 5.01e^-9^ | 0.10 |
| Residual | 0.24 |  |  |  |
| *2018 L. gibbosus (marginal R^2^=0.01, conditional R^2^=019)^†^* | | | | |
| Intercept | 5.36 (0.51) | 34 |  |  |
| Water calcium | -0.001 (0.004) | 2 | 0.21 | 0.65 |
| Body size (L_s_) | -0.03 (0.33) | 34 | 1.02 | 0.32 |
| **Random**  (repeatability)^‡^ | 0.2 |  |  |  |
| Site | 0.08 |  | 0.64 | 0.43 |
| Residual | 0.16 |  |  |  |

^†^Marginal R^2^ describes the proportion of total variance explained by the fixed effects in the model. Conditional R^2^ described the proportion of total variance explained by the fixed effects and the random effect together.

^‡^Repeatability was estimated as the proportion of the remaining variance (not explained by the fixed effects).

##### **TABLE A2** Sample sizes (number of fish analyzed “N”) and geographic coordinates per species per year for each sample site.

| Species | Water Type | Site | Latitude (DD) | Longitude (DD) | N | Year |
| --- | --- | --- | --- | --- | --- | --- |
| *P. caprodes* | Ion poor | BIZ | 45.516119 | -73.897533 | 14 | 2017 |
| *P. caprodes* | Ion poor | GRE | 45.629803 | -74.607868 | 14 | 2017 |
| *P. caprodes* | Ion rich | MEL | 45.319210 | -73.628341 | 12 | 2017 |
| *P. caprodes* | Ion rich | PDC | 45.333996 | -73.961851 | 13 | 2017 |
| *P. caprodes* | Mixed | RAF | 45.415151 | -73.628341 | 6 | 2017 |
| *L. gibbosus* | Ion poor | BIZ | 45.516119 | -73.897533 | 3 | 2017 |
| *L. gibbosus* | Ion poor | GRE | 45.629803 | -74.607868 | 13 | 2017 |
| *L. gibbosus* | Ion rich | MEL | 45.319210 | -73.628341 | 12 | 2017 |
| *L. gibbosus* | Ion rich | PDC | 45.333996 | -73.961851 | 15 | 2017 |
| *L. gibbosus* | Mixed | RAF | 45.415151 | -73.628341 | 13 | 2017 |
| *P. flavescens* | Ion poor | BIZ | 45.516119 | -73.897533 | 13 | 2017 |
| *P. flavescens* | Ion poor | GRE | 45.629803 | -74.607868 | 14 | 2017 |
| *P. flavescens* | Ion rich | MEL | 45.319210 | -73.628341 | 14 | 2017 |
| *P. flavescens* | Ion rich | PDC | 45.333996 | -73.961851 | 13 | 2017 |
| *P. flavescens* | Mixed | RAF | 45.415151 | -73.628341 | 14 | 2017 |
| *P. caprodes* | Ion rich | COT | 45.253611 | -74.211944 | 10 | 2018 |
| *P. caprodes* | Ion poor | GRE | 45.629803 | -74.607868 | 0 | 2018 |
| *P. caprodes* | Ion rich | MSA | 45.096041 | -74.413554 | 10 | 2018 |
| *P. caprodes* | Ion poor | OKA | 45.459560 | -74.087574 | 5 | 2018 |
| *L. gibbosus* | Ion rich | COT | 45.253611 | -74.211944 | 10 | 2018 |
| *L. gibbosus* | Ion poor | GRE | 45.629803 | -74.607868 | 10 | 2018 |
| *L. gibbosus* | Ion rich | MSA | 45.096041 | -74.413554 | 10 | 2018 |
| *L. gibbosus* | Ion poor | OKA | 45.459560 | -74.087574 | 9 | 2018 |
| *P. flavescens* | Ion rich | COT | 45.253611 | -74.211944 | 10 | 2018 |
| *P. flavescens* | Ion poor | GRE | 45.629803 | -74.607868 | 9 | 2018 |
| *P. flavescens* | Ion rich | MSA | 45.096041 | -74.413554 | 10 | 2018 |
| *P. flavescens* | Ion poor | OKA | 45.459560 | -74.087574 | 10 | 2018 |

##### **TABLE A3** Linear mixed effect model predicting scalar calcium concentrations of *P. caprodes*, *L. gibbosus,* and *P. flavescens* along an ionic gradient in the St. Lawrence system. Scalar calcium concentrations and body size were log_10_ transformed before model fitting. Significance of effects were estimated using a log-likelihood ratio test.

| **Effect** | **Estimate** | **d.f.** | **LLR** | **p-value** |
| --- | --- | --- | --- | --- |
| *2017 and 2018 (marginal R^2^=0.58, conditional R^2^=0.61)^†^* | | | | |
| Intercept | 5.43 (0.24) | 272 |  |  |
| SpeciesPS | 0.02 (0.08) | 272 | 34.70 | **<0.0001** |
| SpeciesYP | 0.28 (0.07) | 272 |  |  |
| Water calcium | 0.005 (0.002) | 6 | 8.73 | **0.03** |
| Body size (L_s_) | -0.38 (0.13) | 272 | 8.73 | **0.003** |
| Year | 0.42 (0.05) | 272 | 53.71 | **< 0.0001** |
| SpeciesPS:cond | -0.002 (0.002) | 272 | 6.52 | **0.04** |
| SpeciesYP:cond | -0.005 (0.002) | 272 |  |  |
| **Random effect**  (repeatability)^‡^ | 0.05 |  |  |  |
| Site | 0.05 |  | 7.04 | **0.008** |
| Residual | 0.21 |  |  |  |

^†^Marginal R^2^ describes the proportion of total variance explained by the fixed effects in the model. Conditional R^2^ described the proportion of total variance explained by the fixed effects and the random effect together.

^‡^Repeatability was estimated as the proportion of the remaining variance (not explained by the fixed effect
